# Preoperative black line sign predicts progressive kyphosis after percutaneous kyphoplasty in osteoporotic vertebral compression fractures

**DOI:** 10.1186/s40001-026-03898-9

**Published:** 2026-01-16

**Authors:** Lei He, Yi Hong, Zhenan Chen, Cong Jin, Feiyue Lin

**Affiliations:** 1 College of Clinical Medicine for Oncology, Fujian Medical University, Fuzhou, People’s Republic of China; 2 Department of Orthopaedics, Shaoxing People’s Hospital, Shaoxing, Zhejiang People’s Republic of China; 3 School of Medicine, Shaoxing University, Shaoxing, Zhejiang People’s Republic of China

**Keywords:** Osteoporotic fractures, Kyphosis, Risk factors, Magnetic resonance imaging

## Abstract

**Objectives:**

This study aimed to evaluate the black line sign (BLS) on preoperative MR–STIR images as a predictor of progressive kyphosis (PK) following percutaneous kyphoplasty (PKP) for osteoporotic vertebral compression fractures (OVCFs). The goal of this study was to assess its significance in identifying patients at greater risk for postoperative kyphotic deformities and to refine clinical management strategies.

**Methods:**

This single-center retrospective study analyzed 182 patients with OVCFs who underwent PKP between January 2019 and December 2022. Patients were categorized into two groups based on the presence or absence of BLS on preoperative MRI: the BLS group and the non-black line sign (NBLS) group. Radiological and clinical outcomes were compared between these two groups. Both univariate and multivariate analyses were performed to identify risk factors associated with the development of PK.

**Results:**

At the 1-year follow-up, the BLS group presented significantly greater vertebral height loss and kyphotic angle differences than did the NBLS group. Furthermore, the visual analog scale and Oswestry Disability Index scores were notably higher in the BLS group (*P* < 0.001). The incidence of PK in the BLS group was 75.0% (39/52), which was significantly greater than the 16.2% (21/130) observed in the NBLS group (*P* < 0.001). Cumulative event analysis revealed that a greater proportion of patients in the BLS group developed PK over the follow-up period. Both the log-rank test and Cox regression analysis demonstrated statistically significant differences (*P* < 0.001). Multivariate regression analysis revealed that BLS was a significant independent risk factor for PK, with an odds ratio of 9.827 (*P* < 0.001).

**Conclusions:**

The black line sign on preoperative MR–STIR images is a significant predictor of PK after PKP for OVCFs. Clinicians should consider closer monitoring, external bracing, and aggressive osteoporosis treatment for patients with BLS to reduce the risk of postoperative kyphotic deformities.

**Supplementary Information:**

The online version contains supplementary material available at 10.1186/s40001-026-03898-9.

## Introduction

Osteoporotic vertebral compression fractures (OVCFs) are a common consequence of osteoporosis, leading to significant pain, disability, and diminished quality of life. As the aging population continues to increase globally, the incidence of OVCFs is expected to rise, making it a major health concern [1–3]. Among the treatment options for OVCFs, percutaneous kyphoplasty (PKP) has emerged as a widely used minimally invasive procedure [4, 5]. PKP involves the insertion of a balloon into the vertebral body, which is inflated to restore vertebral height and injected with bone cement to stabilize the fracture [3, 6]. This technique offers significant advantages by providing substantial pain relief, correcting spinal kyphotic deformity, and improving postoperative function [1, 4–6].

Nevertheless, progressive kyphosis (PK) and vertebral recollapse are common complications following PKP, potentially affecting long-term postoperative outcomes by increasing pain and impairing function. The literature reports a vertebral recollapse rate ranging from 11.56% to 38.9% [7, 8], whereas the incidence of PK ranges from 11.2% to 36.4% [9, 10]. Previous studies have identified several factors associated with vertebral recollapse, including low bone mineral density (BMD), intravertebral clefts, cement volume, cement morphology, and a large preoperative kyphotic angle [7, 8, 11, 12]. However, to date, few studies have reported the risk factors for progressive kyphotic deformity following PKP. Understanding these factors is crucial for enhancing treatment strategies and improving patient outcomes.

The black line sign (BLS) on MR STIR images was initially described by Omi, who proposed that this sign may act as a significant marker for identifying high-risk cases of non-union in OVCFs threated conservatively [13]. To date, there is no literature addressing the relationship between MRI signals and postoperative outcomes following PKP. The objective of this study was to investigate the potential of the BLS on MRI as a key predictor of PK after PKP for OVCFs. By evaluating its significance and predictive value, this study aims to provide insights that could help clinicians identify high-risk patients and refine postoperative management strategies.

## Materials and methods

### Study design

This was a single-center retrospective study that analyzed 182 patients with OVCFs who underwent PKP treatment from January 2019 to December 2022. The patients were divided into two groups based on the presence or absence of the BLS on preoperative MR–STIR images: the BLS group and the non-black line sign (NBLS) group. This study compared the radiological and clinical outcomes between the two groups and used univariate and multivariate regression analyses to identify risk factors for PK after PKP surgery, determining whether BLS is a significant risk factor. This study was approved by the Ethics Committee of Shaoxing People's Hospital (2024SRP042-01), and all patients provided informed consent.

### Participants

The inclusion criteria for this study were as follows: patients aged over 60 years who underwent PKP surgery for a single-segment thoracic or lumbar fracture caused by low-energy trauma, with a preoperative BMD ≤ −1.0, a recent fracture confirmed by preoperative MRI T1-weighted imaging, and a follow-up period of over 1 year. The exclusion criteria included a history of previous spinal surgery, secondary osteoporosis due to long-term glucocorticoid use or endocrine disorders, spinal infections, and pathological fractures caused by primary or metastatic spinal tumors. In addition, patients who developed new adjacent-segment vertebral fractures after PKP were excluded to avoid confounding effects.

### Magnetic resonance imaging (MRI) evaluation

**Fig. 1:**
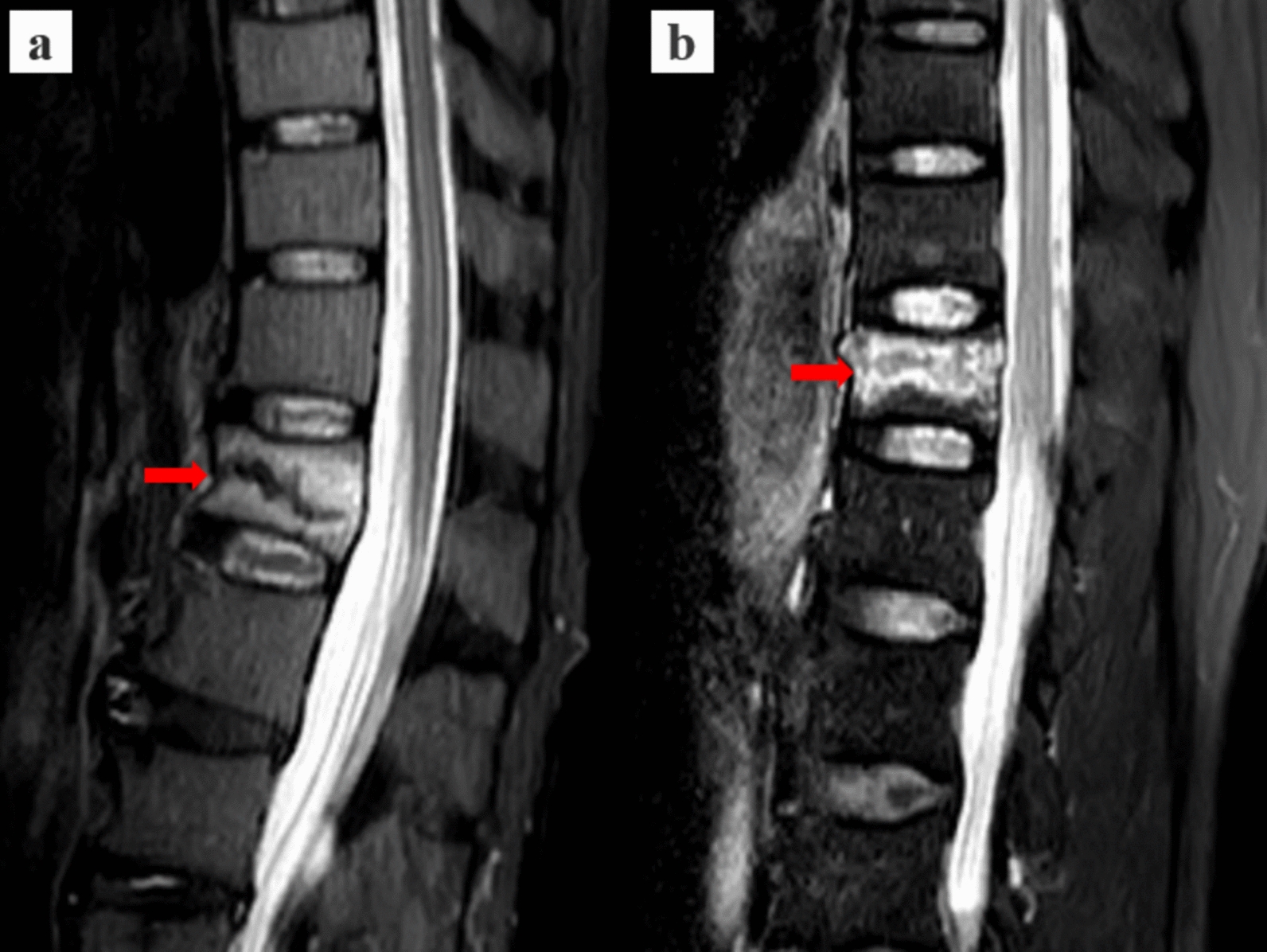
Typical black line sign on MR–STIR images. **a** Black line sign is defined as a signal on the MR–STIR image in which the length of the black line exceeds half of the anterior–posterior diameter of the vertebral body. The typical black line sign is indicated by the red arrow. **b** Non-black line sign is indicated by the red arrow

All patients underwent preoperative spinal MRI using a 1.5 T MRI device (Siemens, Germany) equipped with a spine coil. The examination protocol included T1-weighted sagittal, T2-weighted axial, T2-weighted sagittal, and short tau inversion recovery (STIR) sagittal sequences. Recent osteoporotic vertebral compression fractures were diagnosed based on the presence of a hypointense signal on T1-weighted imaging (T1WI). In addition, the BLS was evaluated on STIR images. The BLS is defined as a black line signal whose length exceeds half of the anterior–posterior diameter of the vertebral body [13] (Fig. 1).

### Percutaneous kyphoplasty

All PKP surgeries were performed by the same surgical team. The patient is positioned prone on a radiolucent table, and the fracture site is identified. The skin over the vertebral body to be treated is then marked. Fluoroscopic guidance is used throughout the procedure to ensure precise needle placement. After administering local anesthesia, a small skin incision was made at the marked point. A hollow needle was inserted through the skin and advanced into the vertebral body under fluoroscopic guidance, reaching the center of the vertebra. Once properly positioned, a specially designed balloon catheter is then introduced, and the balloon is carefully advanced into the fractured vertebra. Once in the correct position, the balloon is inflated with contrast material under fluoroscopic guidance. After the desired vertebral height restoration is achieved, the balloon is deflated and removed, leaving a cavity for the bone cement injection. The prepared cement is then injected into the cavity, with fluoroscopic monitoring to ensure that the cement remains contained within the vertebra and does not leak into surrounding tissues. Once the cement hardens, the small incision is closed with a sterile dressing. The puncture tools and balloon used during the procedure were manufactured by Canwell (Canwell, Jinhua, Zhejiang, China).

Within 24 h after surgery, patients were allowed to wear a brace and begin weight-bearing activities. Postoperatively, short-term external bracing was maintained for approximately 4–6 weeks. All patients received postoperative osteoporosis treatment, including oral Caltrate D 600 mg once daily, oral calcitriol 0.25 µg once daily, and a 4 mg intravenous infusion of zoledronic acid on the first postoperative day, which was administered annually for 3 years.

### Baseline data outcomes

BMD was measured via dual-energy X-ray absorptiometry (DXA), with the BMD value calculated as the average of the lumbar spine (L1–L4) excluding the fractured vertebrae. All baseline data, including age, gender, fracture location, fracture type, height, weight, body mass index (BMI), comorbidities, such as hypertension and diabetes, smoking history, and alcohol consumption history, were retrieved directly from the electronic medical records system.

### Radiographic outcomes

The radiographic evaluation parameters included vertebral height loss (VHL) and the kyphotic angle (KA), with assessment timepoints of preoperative, postoperative, 1 month, 3 months, and 1 year after surgery. VHL and KA were measured on lateral X-ray films. All the radiographic measurements were performed by two senior radiologists, and the results were averaged. Preoperative lateral radiographs were obtained in the supine position due to pain-related intolerance of standing. Postoperative and follow-up lateral radiographs were routinely acquired in the upright standing position.

According to previous studies [9], the vertebral height (VH) is defined as the average of the anterior and posterior vertebral body heights. VHL is calculated as the difference between the average VH of the adjacent vertebrae above and below the fractured vertebra and the VH of the fractured vertebra, divided by the average VH of the adjacent vertebrae above and below, and then multiplied by 100%. The postoperative VHL difference was defined as the preoperative VHL value minus the postoperative VHL value. The VHL recovery rate was calculated by dividing the postoperative VHL difference by the preoperative VHL, then multiplied by 100%. The postoperative follow-up VHL difference was defined as the difference between the postoperative VHL and the postoperative follow-up VHL:

i) VHL = [(Average VH of the adjacent vertebrae above and below)–(VH of the fractured vertebra)]/(Average VH of the adjacent vertebrae above and below) * 100%.
ii) Postoperative VHL difference = Preoperative VHL–Postoperative VHL.
iii) VHL recovery rate = (Preoperative VHL–Postoperative VHL)/Preoperative VHL*100%.
iv) Follow-up VHL difference = Follow-up VHL–Postoperative VHL.

KA is defined as the angle between the superior endplate of the vertebra above the fractured vertebra and the inferior endplate of the vertebra below the fractured vertebra. The postoperative KA difference was calculated as the preoperative KA minus the postoperative KA. The KA recovery rate was calculated by dividing the postoperative KA difference by the preoperative KA. The postoperative follow-up KA difference was defined as the difference between the postoperative KA and the postoperative follow-up KA. PK is defined as a difference greater than 10° between the follow-up KA and the postoperative KA [10].

i) Postoperative KA difference = Preoperative KA–Postoperative KA.
ii) KA recovery rate = (Preoperative KA–Postoperative KA)/Preoperative KA*100%.
iii) Follow-up KA difference = Follow-up KA–Postoperative KA.

### Clinical outcomes

The clinical outcome evaluation parameters included VAS (visual analog scale) [14] and ODI (Oswestry Disability Index) scores [15], with assessment timepoints at the preoperative, postoperative, and 1 year postoperatively. Pain levels were measured via the VAS, where zero represents no pain and a score of 10 indicates intolerable pain. The ODI was used for self-assessment of disability.

### Operative outcomes

Surgical-related data, including operative time, bone cement volume, and puncture approach (unilateral or bilateral), were obtained directly from the surgical records in the electronic medical records system. In accordance with previous studies, the shape of the bone cement was assessed on postoperative lateral X-ray films [16]. Type A: bone cement only contacts the superior endplate. Type B: bone cement only contacts the inferior endplate. Type C: bone cement contacts both the superior and inferior endplates. Type D: bone cement does not contact either the superior or inferior endplate. Postoperative anteroposterior X-ray films were also used to evaluate any bone cement leakage. The locations of bone cement leakage included the intervertebral space, lateral vertebrae, anterior vertebrae, and basivertebral foramen.

### Statistical analysis

Statistical analysis was performed via SPSS (version 19.0; SPSS Inc., Chicago, IL, USA) on the Windows platform. The comparison of gender, fracture type, hypertension history, cement leakage, and PK rates between the two groups were conducted via the chi-square test. Yates' correction was applied to assess fracture location, history of diabetes, smoking, alcohol use, puncture approach, bone cement form, and leakage site between the groups. Independent samples *t* tests were used to evaluate weight, postoperative VHL, VHL at 1 year, and postoperative KA differences, with normality confirmed by the Shapiro‒Wilk test and variance homogeneity assessed by Levene's test. Welch *t* tests were used to compare height and KA at 1 year between the groups. Age, BMI, BMD, preoperative VHL, postoperative VHL difference, VHL recovery rate, VHL difference at 1 year, preoperative KA, postoperative KA, KA recovery rate, KA difference at 1 year, operative time, and bone cement volume between the groups were analyzed using Wilcoxon tests. Two-way mixed ANOVA tests were employed to analyze the VAS and ODI scores. Furthermore, the relationships between PK and the independent variables were modeled via logistic regression. Cumulative curve analysis of PK between the two groups was conducted using both Cox regression and log-rank tests. A significance level of 0.05 was applied.

## Results

### Baseline data

A total of 329 patients were screened. Eighty-three patients were excluded before enrollment, leaving 246 patients who met the inclusion criteria and underwent PKP. During follow-up, 64 patients were excluded, including 42 who developed new adjacent-segment vertebral fractures and 22 with incomplete follow-up data. Finally, 182 patients were included in the analysis (Supplementary Fig. 1).

Of the 182 included patients, 52 were assigned to the BLS group and 130 to the NBLS group. No significant differences were found between the two groups regarding gender, age, height, weight, BMI, BMD, history of hypertension, history of diabetes, smoking, or alcohol use (P > 0.05). However, significant differences in fracture location and fracture type were observed between the two groups (P < 0.001) (Table 1).

**Table 1:** Comparison of baseline data between the two groups

Characteristics	NBLS Group	BLS Group	*P* value
n	130	52	
Gender, n (%)			0.912
Male	31 (23.8%)	12 (23.1%)	
Female	99 (76.2%)	40 (76.9%)	
Age (Y), median (IQR)	72 (67, 76.75)	70 (65, 77)	0.665
Fracture location, n (%)			< 0.001
T	12 (9.2%)	1 (1.9%)	
TL	91 (70.0%)	50 (96.2%)	
L	27 (20.8%)	1 (1.9%)	
Fracture type, n (%)			< 0.001
Compression fracture	120 (92.3%)	34 (65.4%)	
Burst fracture	10 (7.7%)	18 (34.6%)	
Height (m), mean ± SD	1.59 ± 0.08	1.60 ± 0.06	0.830
Weight (kg), mean ± SD	58.79 ± 9.15	57.65 ± 8.68	0.446
BMI (kg/m^2^), median (IQR)	23.00 (20.77, 24.75)	22.49 (20.96, 23.76)	0.375
BMD, median (IQR)	−3.0 (−3.8, −2.1)	−3 (−3.7, −1.9)	0.701
Hypertension, n (%)			0.372
Yes	58 (44.6%)	27 (51.9%)	
No	72 (55.4%)	25 (48.1%)	
Diabetes, n (%)			0.264
Yes	9 (6.9%)	7 (5.4%)	
No	121 (93.1%)	45 (94.6%)	
Smoking, n (%)			1.000
Yes	4 (3.1%)	1 (1.9%)	
No	126 (96.9%)	51 (98.1%)	
Alcohol, n (%)			0.055
Yes	7 (5.4%)	8 (15.4%)	
No	123 (94.6%)	44 (84.6%)	

NBLS, non-black line sign; BLS, black line sign; T, thoracic spine; L, lumbar spine; TL, thoracolumbar spine; BMD, bone mineral density; BMI, body mass index; IQR, interquartile range; SD, standard deviation.

### Radiographic outcomes

Compared with the NBLS group, the BLS group had significantly greater preoperative VHL (*P* < 0.001). Postoperatively, the BLS group exhibited greater VHL (*P* = 0.036) and a significantly greater VHL difference (*P* = 0.001), as well as a higher VHL recovery rate (*P* = 0.038), indicating better initial restoration of vertebral height. However, at 1 year postoperatively, the BLS group demonstrated significantly greater VHL than the NBLS group did (*P* < 0.001) and a significantly greater VHL difference (*P* = 0.002), suggesting greater vertebral height loss and less effective long-term maintenance of vertebral height (Table 2).

**Table 2:** Comparison of vertebral height loss and kyphotic angle between the two groups

Characteristics	NBLS Group	BLS Group	*P* value
VHL at preop (%), median (IQR)	12.55 (7.10, 19.05)	19.45 (15.10, 26.95)	< 0.001
VHL at postop (%), mean ± SD	11.53 ± 9.79	14.83 ± 8.89	0.036
VHL difference at postop (%), median (IQR)	0.95 (−2.30, 5.80)	3.80 (1.60, 10.78)	0.001
VHL recovery rate (%), median (IQR)	7.13 (−19.68, 47.19)	22.04 (9.09, 45.99)	0.038
VHL at 1 year (%), mean ± SD	13.85 ± 10.18	20.19 ± 9.66	< 0.001
VHL difference at 1 year (%), median (IQR)	1.20 (−0.60, 4.98)	4.80 (0.55, 9.08)	0.002
KA at preop (°), median (IQR)	13.65 (1.80, 22.88)	21.45 (13.05, 29.85)	< 0.001
KA at postop (°), median (IQR)	12.05 (0.49, 22.58)	14.30 (10.23, 26.45)	0.019
KA difference at postop (°), mean ± SD	1.17 ± 4.65	4.24 ± 5.03	< 0.001
KA recovery rate (%), median (IQR)	3.31 (−12.67, 19.93)	19.43 (4.35, 34.52)	< 0.001
KA at 1 year (°), mean ± SD	12.65 ± 18.71	26.91 ± 11.72	< 0.001
KA difference at 1 year (°), median (IQR)	1.30 (−0.90, 4.18)	10.80 (9.30, 12.33)	< 0.001

VHL: vertebral height loss; KA: kyphotic angle; NBLS: non-black line sign; BLS: black line sign; IQR: interquartile range; SD: standard deviation.

The BLS group had significantly higher preoperative KA compared to the NBLS group (*P* < 0.001). Postoperatively, the BLS group presented a significantly greater KA difference and KA recovery rate (*P* < 0.001), indicating better initial correction of kyphotic deformity after surgery. However, at 1 year postoperatively, the BLS group had significantly greater KA and KA difference than the NBLS group did (*P* < 0.001), suggesting less effective long-term correction of the kyphotic deformity (Table 2).

### Operative outcomes

There were no significant differences between the NBLS and BLS groups in terms of operative time (P = 0.066). The median bone cement volume was 6.0 mL (IQR 5.6–6.0 mL; range 3.0–12.0 mL) in the NBLS group and 6.0 mL (IQR 6.0–6.0 mL; range 3.0–9.0 mL) in the BLS group (P = 0.571). Most patients in both groups received bilateral punctures, with no significant difference in the type of puncture used (P = 0.844). In addition, no significant differences were observed between the groups regarding bone cement form (P = 0.677). Cement leakage occurred in 44.6% of patients in the NBLS group and 44.2% in the BLS group, with this difference not being statistically significant (P = 0.962). The distribution of leakage sites also showed no significant difference between the groups (P = 0.449) (Table 3).

**Table 3:** Comparison of surgery-related outcomes between the two groups

Characteristics	NBLS Group	BLS Group	*P* value
Operative time (min), median (IQR)	34 (30, 42)	30 (24, 39)	0.066
Bone cement volume (ml), median (IQR)	6 (5.625, 6)	6 (6, 6)	0.571
Puncture approach, n (%)			0.844
Unilateral	5 (3.8%)	1 (1.9%)	
Bilateral	125 (96.2%)	51 (98.1%)	
Bone cement form, n (%)			0.677
A	10 (7.7%)	3 (5.8%)	
B	31 (23.8%)	9 (17.3%)	
C	80 (61.5%)	37 (71.2%)	
D	9 (6.9%)	3 (5.8%)	
Cement leakage, n (%)			0.962
Yes	58 (44.6%)	23 (44.2%)	
No	72 (55.4%)	29 (55.8%)	
Leakage site, n (%)			0.449
No leakage	72 (55.4%)	28 (53.8%)	
Intervertebral space	10 (7.7%)	3 (5.8%)	
Lateral vertebrae	15 (11.5%)	8 (15.4%)	
Anterior vertebrae	16 (12.3%)	10 (19.2%)	
Basivertebral foramen	17 (13.1%)	3 (5.8%)	

NBLS: non-black line sign; BLS: black line sign.

### Clinical outcomes

**Fig. 2:**
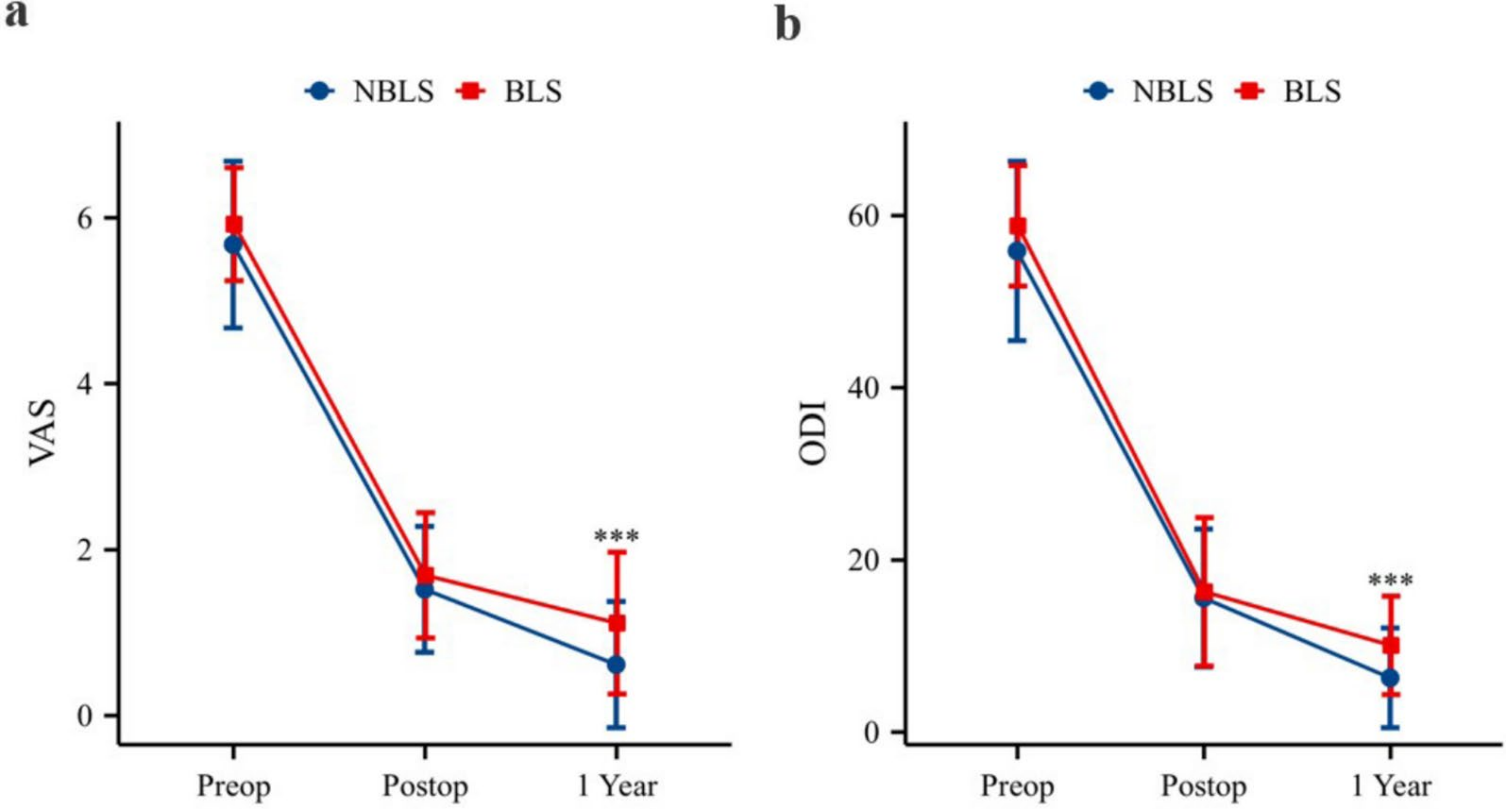
Comparison of clinical outcomes between the two groups. **a** Comparison of VAS scores between the two groups. **b** Comparison of the ODI scores between the two groups. ***: P < 0.001, BLS group compared with the NBLS group. NBLS: non-black line sign; BLS: black line sign; VAS: visual analog scale; ODI: Oswestry Disability Index.

The comparison of VAS scores between the BLS and NBLS groups before and after surgery revealed no significant difference. However, 1 year after surgery, the VAS score in the BLS group was significantly greater than that in the NBLS group (P < 0.001) (Fig. 2a).

Similarly, there was no significant difference in the ODI score between the two groups before and after surgery. However, 1 year postoperatively, the ODI score in the BLS group was significantly greater than that in the NBLS group (P < 0.001) (Fig. 2b).

### Progressive kyphosis rates and cumulative event analysis

**Fig. 3:**
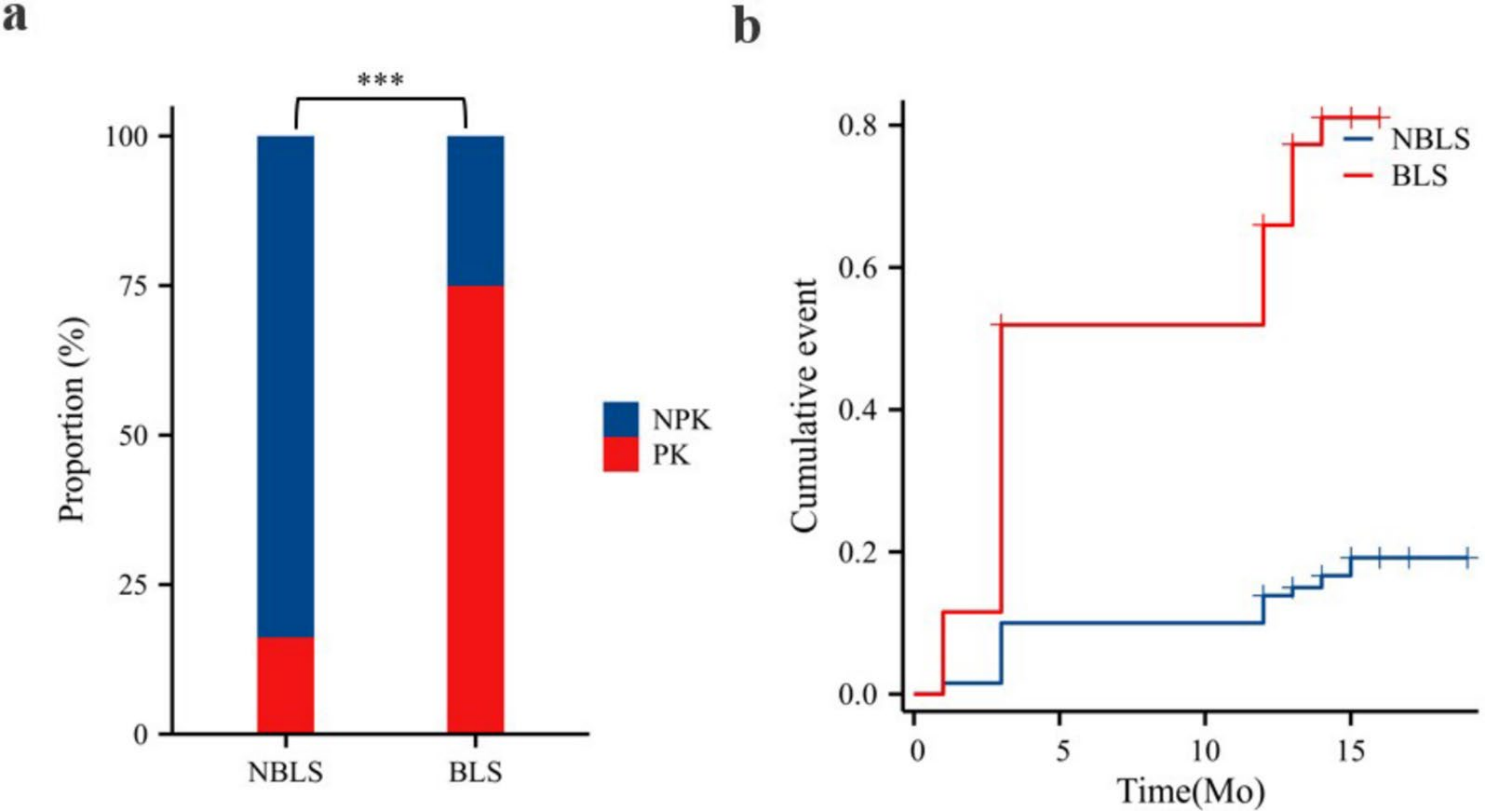
Comparison of progressive kyphosis rates and cumulative event analysis between the two groups. **a** Comparison of progressive kyphosis rates between the two groups. **b** Cumulative event analysis of progressive kyphosis in the two groups. NBLS: non-black line sign; BLS: black line sign; PK: progressive kyphosis; NPK: non-progressive kyphosis. ***: P < 0.001, BLS group compared with the NBLS group.

One year after surgery, 60 out of 182 patients developed PK, resulting in an overall PK rate of 33.0%. In the BLS group, the PK rate was 75.0% (39/52), which was significantly greater than the 16.2% (21/130) in the NBLS group (P < 0.001) (Fig. 3a).

Cumulative event analysis indicated that the BLS group had a greater proportion of patients who developed PK over the follow-up period. Both the log-rank test and Cox regression analysis revealed significant differences (P < 0.001) (Fig. 3b).

### Univariate analysis of risk factors for progressive kyphosis

In the univariate analysis, BLS (OR = 15.571, 95% CI 7.121–34.051, P < 0.001), Burst fracture (OR = 7.125, 95% CI 2.910–17.447, P < 0.001), preoperative VHL (OR = 1.046, 95% CI 1.015–1.078, P = 0.003), preoperative KA (OR = 1.079, 95% CI 1.047–1.111, P < 0.001) and postoperative KA (OR = 1.052, 95% CI 1.026–1.079, P < 0.001) were identified as significant risk factors for PK after PKP (Table 4).

**Table 4:** Univariate analysis of risk factors for progressive kyphosis

Characteristics	Univariate analysis		Characteristics	Univariate analysis	
	Odds Ratio (95% CI)	*P* value		Odds Ratio (95% CI)	*P* value
BLS			VHL at preop	1.046 (1.015–1.078)	0.003
No	Reference		VHL at postop	1.015 (0.983–1.048)	0.373
Yes	15.571 (7.121–34.051)	< 0.001	VHL recovery rate	1.001 (0.999–1.003)	0.424
Gender			KA at preop	1.079 (1.047–1.111)	< 0.001
Male	Reference		KA at postop	1.052 (1.026–1.079)	< 0.001
Female	1.584 (0.735–3.415)	0.241	KA recovery rate	1.000 (0.999–1.002)	0.590
Age	1.015 (0.975–1.058)	0.461	Operative time	0.980 (0.951–1.009)	0.177
BMI	0.978 (0.881–1.085)	0.669	Bone cement volume	0.913 (0.758–1.100)	0.339
BMD	0.998 (0.765–1.302)	0.990	Puncture approach		
Fracture type			bilateral	Reference	
Compression fracture	Reference		Unilateral	1.017 (0.181–5.716)	0.985
Burst fracture	7.125 (2.910–17.447)	< 0.001	Bone cement form		
Hypertension			C	Reference	
Yes	Reference		B	0.482 (0.210–1.106)	0.085
No	0.608 (0.326–1.133)	0.117	A	0.498 (0.130–1.907)	0.309
Diabetes			D	0.830 (0.236–2.916)	0.771
No	Reference		Cement leakage		
Yes	2.192 (0.780–6.162)	0.137	Yes	Reference	
Smoking			No	0.794 (0.427–1.477)	0.467
No	Reference		VAS score at preop	1.231 (0.880–1.723)	0.225
Yes	1.368 (0.222–8.413)	0.735	ODI score at preop	1.015 (0.983–1.048)	0.373
Alcohol					
No	Reference				
Yes	2.527 (0.870–7.339)	0.088			

NBLS: non-black line sign; BLS: black line sign; VHL: vertebral height loss; KA: kyphotic angle; VAS: visual analog scale; ODI: Oswestry Disability Index.

### Multivariate analysis of risk factors for progressive kyphosis

**Fig. 4:**
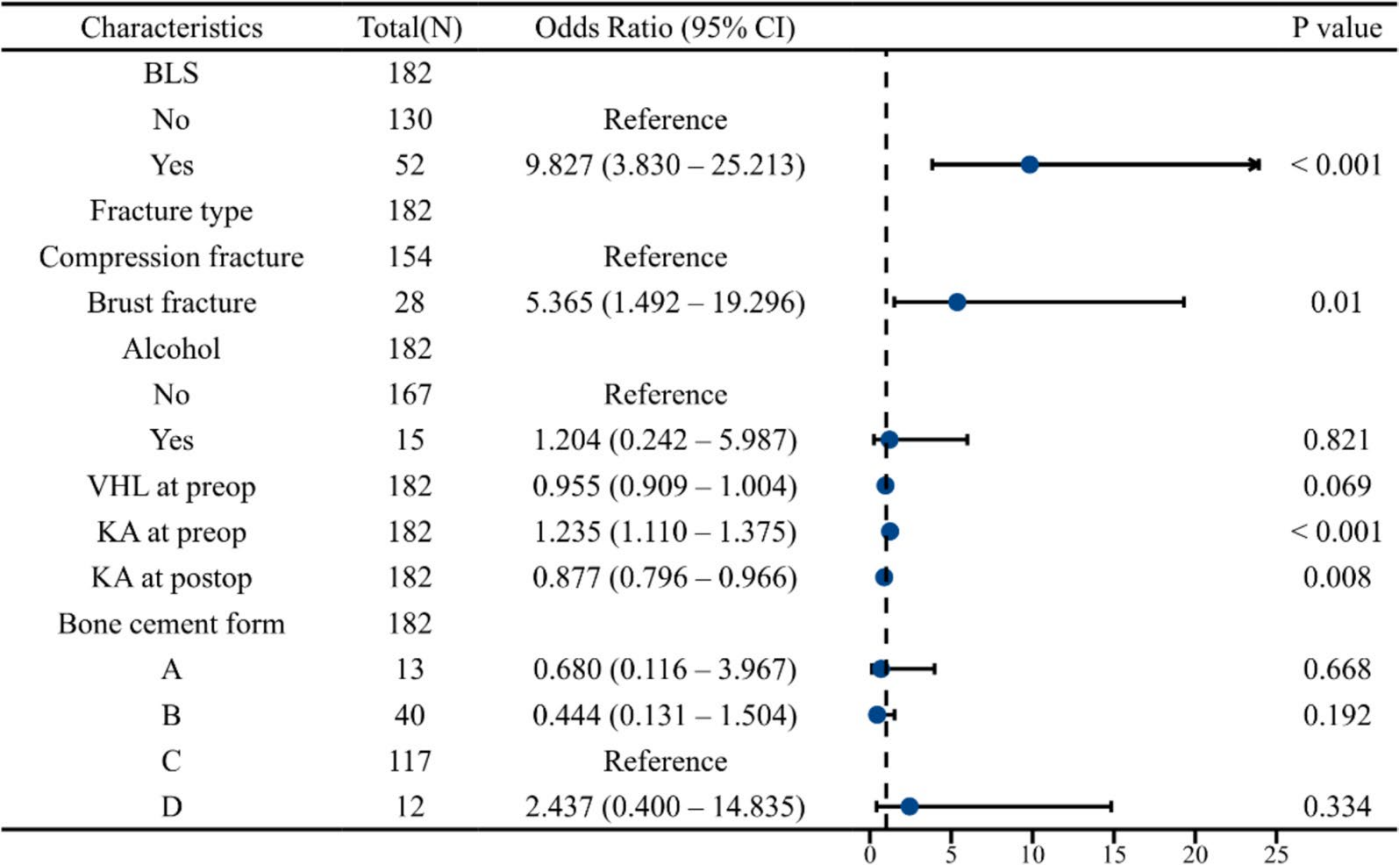
Multivariate analysis of risk factors for progressive kyphosis after PKP. BLS: black line sign; VHL: vertebral height loss; KA: kyphotic angle

In the multivariate analysis, the OR for BLS was 9.827 (95% CI 3.830–25.213), with a *P* value < 0.001, indicating a strong and statistically significant association with an increased risk of PK. In addition, Burst fracture (OR = 5.365, 95% CI 1.492–19.296, P = 0.010), preoperative KA (OR = 1.235, 95% CI 1.110–1.375, P < 0.001), and postoperative KA (OR = 0.877, 95% CI 0.796–0.966, P = 0.008) were identified as significant risk factors for PK after PKP (Fig. 4).

### Evaluation of predictive models for progressive kyphosis

**Fig. 5:**
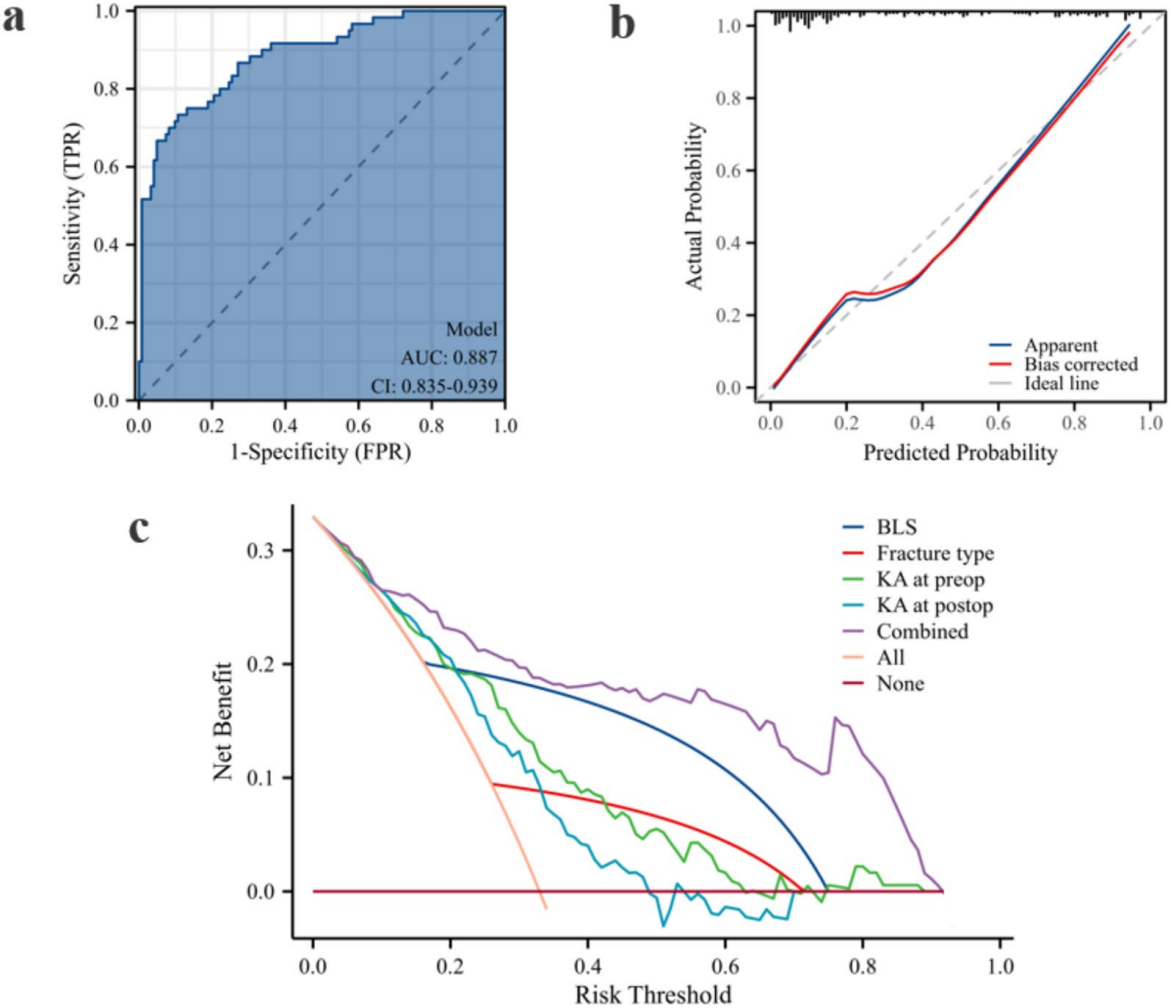
Evaluation of predictive models for progressive kyphosis. (**a**) Receiver operating characteristic curve; (**b**) calibration curve; (**c**) decision curve analysis curve

**Fig. 6:**
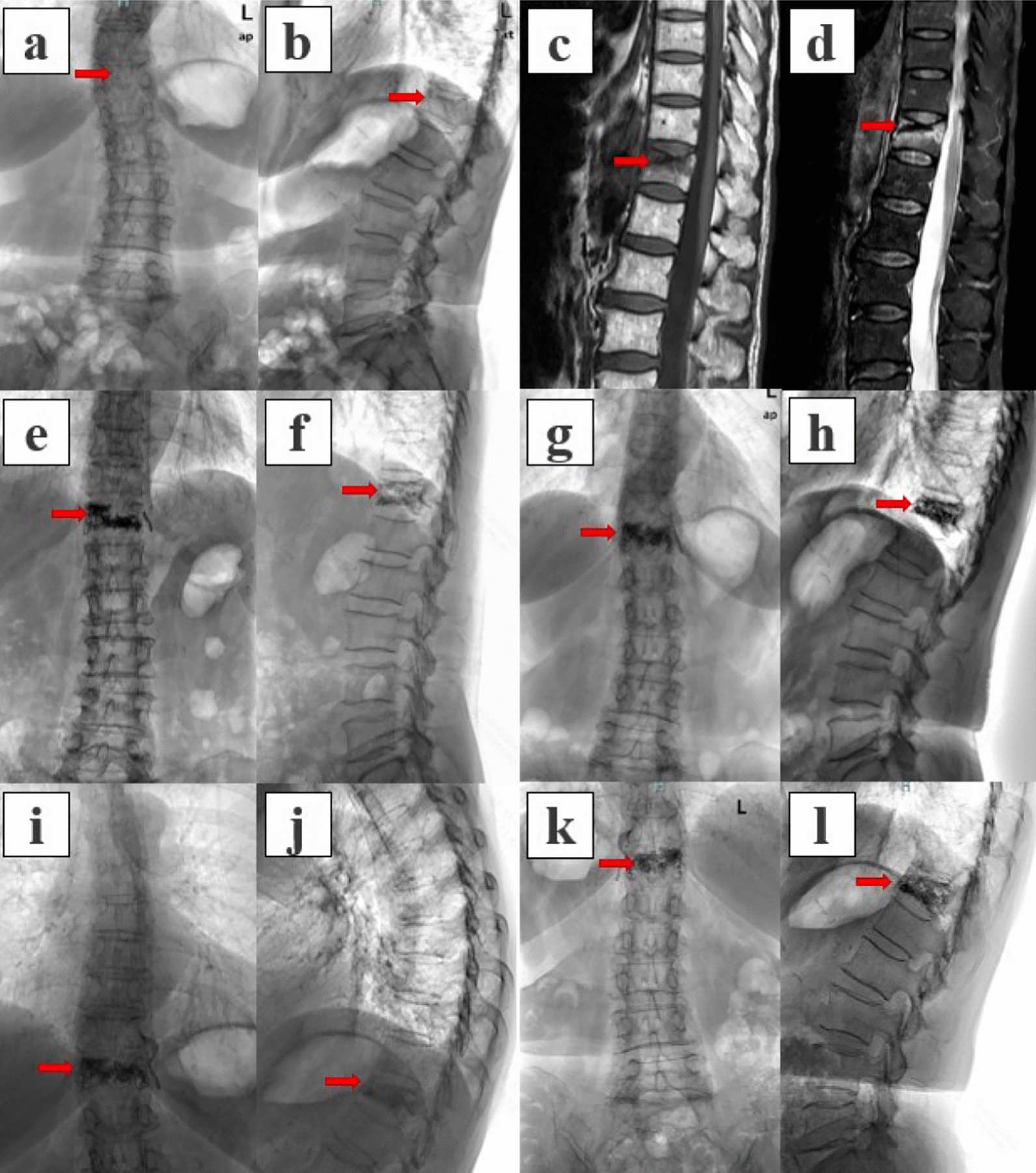
Typical case of the black-line sign group. A 77-year-old female patient presented with a compression fracture of T12 due to low-energy trauma. **a**, **b** Preoperative X-rays showing the T12 compression fracture, indicated by the red arrow. The preoperative VHL was 17.7%, and the KA was 20.8°. **c** Preoperative T1-weighted MR image showing a hypointense signal, confirming a recent fracture at T12. **d** Preoperative MR–STIR images revealing the typical black line sign. **e**, **f** Postoperative X-rays showing good cement dispersion. The postoperative VHL was 5.4%, and the KA was 7.7°. The VHL difference was 12.3%, with a VHL recovery rate of 69.5%, indicating excellent vertebral height restoration postoperatively. The KA difference was 13.1°, with a KA recovery rate of 63.0%, suggesting excellent correction of the kyphotic deformity. **g**, **h** One month postoperatively, the VHL angle was 16.3%, the KA was 15.5°, the VHL difference was 9.9%, and the KA difference was 7.8°, indicating some degree of vertebral height loss and worsening of the kyphotic deformity. **i**, **j** Three months postoperatively, the VHL was 17.2%, the KA was 21.3°, the VHL difference was 11.8%, and the KA difference was 13.6°, indicating progressive kyphotic deformity. **k**, **l** One year after surgery, the fracture had healed, the VHL was 18.1%, the KA was 22.7°, the VHL difference was 12.7%, and the KA difference was 15.0°, suggesting progressive kyphosis. VHL: Vertebral height loss; KA: Kyphotic angle.

The area under the curve (AUC) was 0.887 (95% CI 0.835–0.939), indicating excellent discrimination (Fig. 5a). The calibration curve indicates good agreement between the predicted and actual probabilities (Fig. 5b). The DCA curve revealed that the combined model offered the greatest net benefit, suggesting that it is the most clinically useful for predicting PK (Fig. 5c) (Fig. 6).

## Discussion

The key finding of this study is that BLS on MR STIR images is a significant risk factor for PK following PKP in patients with OVCFs. Patients with BLS had a significantly higher incidence of PK, with a rate of 75.0%, than the 16.2% in the NBLS group. Cumulative event analysis further confirmed that the rate of PK in the BLS group was significantly greater than that in the NBLS group, with both Cox regression and log-rank tests revealing statistically significant differences. In addition, the presence of the BLS was strongly associated with greater VHL and KA differences at both the 1-year follow-up stages. Furthermore, both univariate and multivariate regression analyses identified BLS as a high-risk factor for PK. These findings suggest that the BLS may serve as a valuable radiological marker for identifying patients at high risk of developing progressive deformities after PKP surgery.

The BLS was first reported by Omi, who proposed that it is a high-risk factor for non-union of OVCFs treated conservatively [13]. The BLS may be caused by early osteonecrosis at the fracture site, where the necrotic bone tissue cannot be repaired due to insufficient blood supply, forming a low-signal area that appears as a black line on MRI. The BLS may also indicate severe trabecular damage. When the trabeculae are subjected to significant compression or destruction during the fracture process, the damaged trabeculae may not recover or repair, leading to long-term instability of the bone tissue. This instability further hinders the formation of new bone, potentially resulting in non-union of the fracture. In addition, the BLS may be associated with intravertebral vacuum clefts, which have been reported in the literature as high-risk factors for fracture non-union and vertebral collapse after vertebroplasty [7, 8, 17, 18]. However, these pathophysiological mechanisms remain hypothetical, and future research should focus on investigating the underlying pathological basis of the BLS to better understand its role in the progression of osteoporotic fractures and their outcomes.

Patients with BLS who undergo PKP are more prone to vertebral recollapse and progressive kyphosis after surgery for several potential reasons. BLS typically indicates severe trabecular damage or osteonecrosis, leading to structural instability at the fracture site. This instability weakens the mechanical support of the vertebra, and even when bone cement is injected during kyphoplasty to reinforce the vertebra, the effect of cement fixation may be insufficient, leading to a greater risk of recollapse. Second, in patients with severe trabecular damage, the filling of bone cement may not fully restore the original structure or stability of the bone. Damage to trabeculae leads to inadequate support within the vertebral body, especially when cement filling is uneven or the extent of trabecular damage is extensive, increasing the risk of recollapse. Furthermore, in patients with BLS, it usually indicates significant osteonecrosis or trauma, which reduces the ability of the bone to heal. Even after kyphoplasty, the fracture site may not heal or stabilize effectively due to insufficient bone repair capacity. This lack of biological repair makes the fracture site more susceptible to stress and recollapses after surgery. Finally, the effectiveness of PKP largely depends on the bonding strength between the bone cement and the vertebral bone. If the bone has become fragile due to trabecular damage or osteonecrosis, the adhesive strength of the cement may be weakened, failing to provide adequate support and resulting in vertebral recollapse.

In this study, we also found that burst fracture is a high-risk factor for PK after PKP. Previous research by Jin et al. also considered fracture type as a factor, but their results did not suggest that burst fracture was associated with PK after PKP [9]. Lu et al. reported that fractures of the vertebral posterior wall (OR = 14.16, P < 0.001) are a risk factor for vertebral recollapse [19]. Compression fractures primarily affect the anterior column of the vertebral body, whereas burst fractures commonly accompany fractures of the middle column and posterior wall. Compared with compression fractures, burst fractures lead to greater spinal instability, thus increasing the risk of postoperative PK [4]. In addition, burst fractures may cause more severe trabecular bone damage than compression fractures do, which could result in incomplete filling of the fracture sites by bone cement. This incomplete cement distribution reduces stability and load-bearing capacity, potentially contributing to the development of postoperative PK.

A greater preoperative KA is a recognized risk factor for PK after PKP, which is consistent with the findings of previous studies [9]. Jin’s research also revealed that greater preoperative KA (OR = 1.26, P = 0.008) was a risk factor for PK following PKP [9]. In this study, univariate analysis revealed that greater preoperative vertebral height loss (VHL) (OR = 1.046, P = 0.003) was associated with an increased risk of progressive kyphosis after PKP, although multivariate analysis did not identify it as a significant risk factor (P = 0.069), which aligns with Jin's findings [9]. A larger preoperative KA may lead to a shift in the spinal mechanical axis, causing the vertebrae to bear greater mechanical stress, thus increasing the risk of further deformation and collapse of the vertebrae after surgery, ultimately exacerbating the kyphotic deformity. Wang et al. reported that greater recovery of vertebral height and kyphotic angle postoperatively are high-risk factors for recurrent kyphosis [10]. In this study, these factors were also considered, but they were not confirmed as risk factors in the univariate regression analysis. In addition, as shown in Supplementary Fig. 2, both KA and KA difference at 1 year showed weak correlations with VAS and ODI, which is consistent with previous reports indicating that postoperative correction loss has only limited impact on clinical symptoms [20, 21].

In the present study, although the injected bone cement volume varied within a relatively wide range (3.0–12.0 mL in the NBLS group and 3.0–9.0 mL in the BLS group), there was no significant difference in median cement volume between the two groups. Moreover, additional correlation analyses demonstrated that bone cement volume was not significantly associated with vertebral height restoration or subsequent height loss. As shown in Supplementary Fig. 3, cement volume showed no significant correlation with VHL improvement rate or VHL difference at 1 year in either the NBLS group (ρ = 0.093, P = 0.293; ρ = 0.015, P = 0.867) or the BLS group (ρ = –0.022, P = 0.877; ρ = –0.131, P = 0.356). These findings suggest that, within the range of cement volumes used in our cohort, the absolute amount of cement injected had little influence on vertebral height restoration or recollapse. Thus, the increased risk of progressive kyphosis observed in patients with BLS appears to be more closely related to the underlying vertebral pathology indicated by the BLS itself rather than to differences in bone cement volume.

Both Li and Dong et al. reported that low BMD is a risk factor for vertebral recollapse [7, 11]. However, in this study, low BMD was not identified as a risk factor for PK, which aligns with the findings of Wang [10]. This discrepancy may be due to the primary focus of the studies. While Li and Dong et al. investigated vertebral recollapse postoperatively [7, 11], this study specifically focused on PK. These two outcomes may be influenced by different factors, which could explain why low BMD was associated with recollapse but not with PK in this study.

The key finding of this study, which identified BLS on preoperative MR–STIR images as a significant risk factor for PK after PKP, has important clinical implications. Recognizing the presence of BLS prior to surgery could help clinicians identify patients at higher risk for postoperative complications, such as PK and vertebral recollapse. When the BLS is observed, clinical management strategies may need to be adjusted to mitigate these risks and improve long-term outcomes. First, external bracing with a rigid orthosis may be beneficial to provide additional spinal support, and in our practice is maintained for approximately 8–12 weeks. Second, given the higher likelihood of progressive kyphosis in this subgroup, we advocate closer radiographic surveillance, with follow-up imaging at 1, 2, 3, 6, and 12 months after PKP to monitor vertebral height and kyphotic angle, particularly within the 3 months postoperatively. Third, aggressive postoperative osteoporosis treatment is essential to strengthen the vertebral structure and reduce the risk of subsequent fractures or deformity; in appropriate candidates, intensive regimens including parenteral agents such as denosumab or teriparatide are initiated early. These recommendations are based on the strong association between BLS and PK observed in our cohort, together with extrapolation from current osteoporosis management principles [22–24]. However, direct evidence supporting a dedicated treatment protocol specifically for BLS-positive patients is not yet available, and future prospective studies are needed to validate and refine these management strategies.

While this study provides valuable insights, several limitations should be acknowledged. First, this was a single-center retrospective study, which may limit the generalizability of our findings. Larger multicenter prospective studies are warranted to provide more robust evidence. Second, the follow-up period was limited to 1 year. Although most cases of progressive kyphosis in our cohort occurred within this timeframe, longer follow-up is needed to better characterize the durability of radiographic correction and long-term patient-reported outcomes in BLS-positive patients. Third, adjacent vertebral fractures are a common complication after PKP and may contribute to kyphotic progression. These cases were excluded in the present study to ensure that postoperative deformity was attributable to the treated vertebra itself. This approach improves internal validity but limits external applicability. Future studies should examine whether the presence of BLS increases the risk of secondary or adjacent vertebral fractures after PKP. Moreover, because VHL was normalized using adjacent vertebral reference values, subtle adjacent endplate deformation that does not meet diagnostic criteria for an adjacent fracture could still influence follow-up VHL differences, even though radiographically confirmed adjacent fractures were excluded. Future studies incorporating CT-based assessment of adjacent endplate morphology may help address this issue. Fourth, radiographic measurements may have been affected by patient positioning. Preoperative lateral radiographs were acquired in the supine position due to pain-related intolerance of standing, whereas postoperative and follow-up radiographs were obtained in the upright standing position. Although our primary analyses focused on postoperative-to-follow-up changes measured under the same standing condition, which likely minimized the impact on the main findings, some degree of positioning-related bias cannot be completely excluded. In addition, paired standing and supine lateral radiographs were not routinely available in this retrospective cohort. Therefore, dynamic stability based on position-related differences in VHL and KA, which may be clinically informative after vertebral augmentation, could not be evaluated. Future prospective studies incorporating standardized paired standing and supine imaging are warranted. Finally, although BLS may reflect impaired local healing capacity or avascular changes within the vertebral body, this interpretation remains speculative, because direct histopathological or perfusion-based imaging evidence is currently lacking. Future studies incorporating dynamic contrast-enhanced MRI, nuclear imaging, or histological evaluation in revision cases, as well as biomechanical and molecular investigations, are needed to clarify the biological substrate of BLS and strengthen the mechanistic basis for its association with progressive kyphosis.

## Conclusions

The black line sign on preoperative MR–STIR images is a significant predictor of PK after PKP for OVCFs. Clinicians should consider closer monitoring, external bracing, and aggressive osteoporosis treatment for patients with BLS to reduce the risk of postoperative kyphotic deformities.

## Supplementary Information

Supplementary material 1: Supplementary Figure 1. Patient selection flow diagram.

Supplementary material 2: Supplementary Figure 2. Correlations between KA parameters and clinical outcomes at 1 year after PKP.Scatterplot showing the relationship between the KA at 1 year and VAS scores in the NBLS and BLS groups.Scatterplot illustrating the correlation between KA difference at 1 year and VAS scores.Scatterplot showing the association between KA at 1 year and ODI scores.Scatterplot demonstrating the relationship between KA difference at 1 year and ODI scores. Each dot represents an individual vertebra. Linear trend lines with 95% confidence bands are presented for visualization. Spearman correlation coefficientsand corresponding P values are shown within each panel. Across all analyses, the correlations were weak in magnitude, indicating that kyphotic deformity and its progression had only a limited impact on pain and functional outcomes at 1 year. KA: Kyphotic angle

Supplementary material 3: Supplementary Figure 3. Correlations between bone cement volume and VHL parameters in the NBLS and BLS groups.Scatterplots showing the relationship between bone cement volume and VHL improvement rate Postoperatively.Scatterplots showing the relationship between bone cement volume and VHL difference at 1-year follow-up. Each dot represents a treated vertebra. Lines represent fitted linear trends with 95% confidence bands. Spearman’s correlation coefficientsand corresponding P values for each group are shown within each panel. VHL: Vertebral height loss

## Data Availability

The datasets generated during and/or analyzed during the current study are available from the corresponding author on reasonable request.

## Abbreviations

BLS: Black line sign
PK: Progressive kyphosis
PKP: Percutaneous kyphoplasty
OVCFs: Osteoporotic vertebral compression fractures
BMD: Bone mineral density
NBLS: Non-black line sign
STIR: Short tau inversion recovery
T1WI: T1-weighted images
BMI: Body mass index
VHL: Vertebral height loss
KA: Kyphotic angle
VH: Vertebral height
VAS: Visual analog scale
ODI: Oswestry Disability Index
IQR: Interquartile range
SD: Standard deviation
AUC: Area under the curve
DCA: Decision curve analysis
