# Supplementary figures and images for: Preoperative black line sign predicts progressive kyphosis after percutaneous kyphoplasty in osteoporotic vertebral compression fractures
Source: Eur J Med Res. 2026 Jan 16;31:278. doi: 10.1186/s40001-026-03898-9 (PMC12892439; doi:10.1186/s40001-026-03898-9)

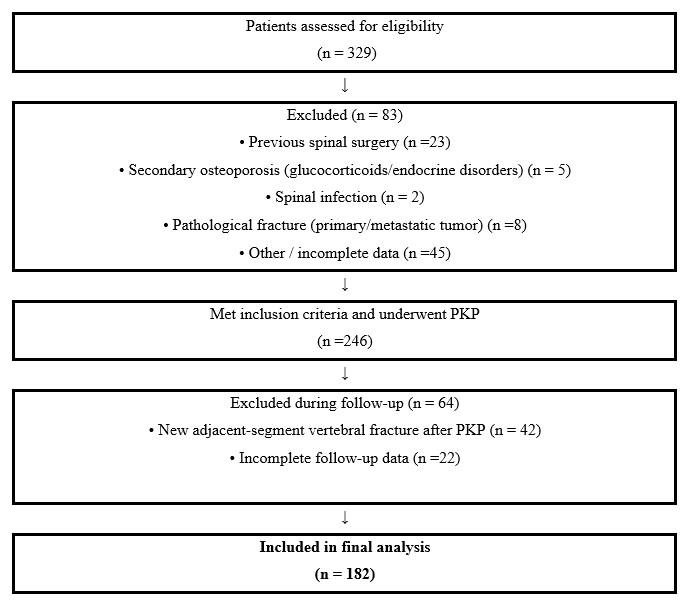

Supplement: Supplementary file 1 — Supplementary material 1: Supplementary Figure 1. Patient selection flow diagram. [file 40001_2026_3898_MOESM1_ESM.tif]

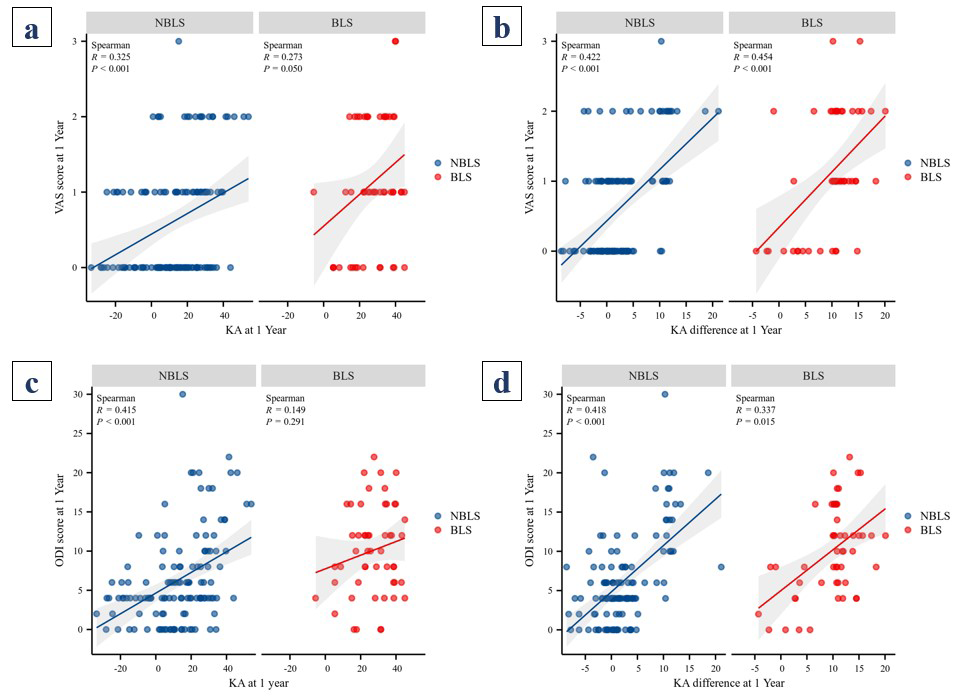

Supplement: Supplementary file 2 — Supplementary material 2: Supplementary Figure 2. Correlations between KA parameters and clinical outcomes at 1 year after PKP.Scatterplot showing the relationship between the KA at 1 year and VAS scores in the NBLS and BLS groups.Scatterplot illustrating the correlation between KA difference at 1 year and VAS scores.Scatterplot showing the association between KA at 1 year and ODI scores.Scatterplot demonstrating the relationship between KA difference at 1 year and ODI scores. Each dot represents an individual vertebra. Linear trend lines with 95% confidence bands are presented for visualization. Spearman correlation coefficientsand corresponding P values are shown within each panel. Across all analyses, the correlations were weak in magnitude, indicating that kyphotic deformity and its progression had only a limited impact on pain and functional outcomes at 1 year. KA: Kyphotic angle [file 40001_2026_3898_MOESM2_ESM.tif]

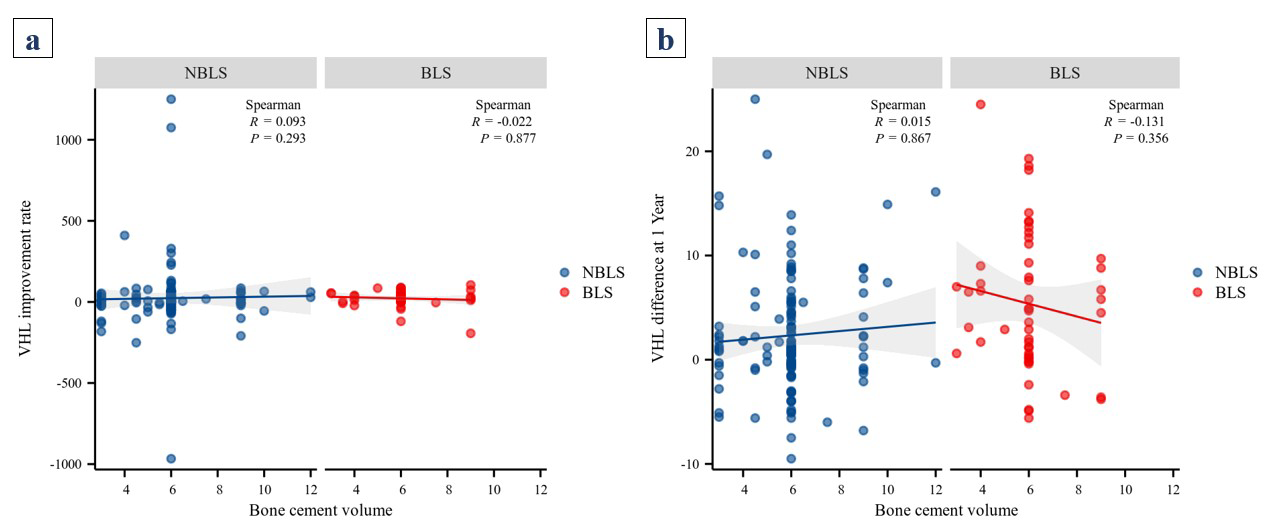

Supplement: Supplementary file 3 — Supplementary material 3: Supplementary Figure 3. Correlations between bone cement volume and VHL parameters in the NBLS and BLS groups.Scatterplots showing the relationship between bone cement volume and VHL improvement rate Postoperatively.Scatterplots showing the relationship between bone cement volume and VHL difference at 1-year follow-up. Each dot represents a treated vertebra. Lines represent fitted linear trends with 95% confidence bands. Spearman’s correlation coefficientsand corresponding P values for each group are shown within each panel. VHL: Vertebral height loss [file 40001_2026_3898_MOESM3_ESM.tif]
